# Artificial Neural Network for Automated Keratoconus Detection Using a Combined Placido Disc and Anterior Segment Ocular Coherence Tomography Topographer

**DOI:** 10.1167/tvst.13.4.13

**Published:** 2024-04-08

**Authors:** Jorge L. Alió del Barrio, Alaa M. Eldanasoury, Juan Arbelaez, Stefano Faini, Francesco Versaci

**Affiliations:** 1 Cornea, Cataract and Refractive Surgery Unit, Vissum (Miranza Group), Alicante, Spain; 2 Division of Ophthalmology, School of Medicine, Universidad Miguel Hernández, Alicante, Spain; 3 Magrabi Eye Hospital, Jeddah, Saudi Arabia; 4 Muscat Eye Laser Center, Muscat, Oman; 5 CSO, Florence, Italy

**Keywords:** MS39, keratoconus, screening, machine learning, automated classification system, artificial neural network, corneal epithelium, OCT, topography

## Abstract

**Purpose:**

To assess the efficacy of an automated program for keratoconus and keratoconus suspect detection based on corneal measurements provided by a combined Placido disc and anterior segment optical coherence tomography (OCT) topographer.

**Methods:**

In a multicentric cross-sectional study, an artificial neural network (ANN) was created using 6677 eyes from an equal number of patients (classified as 2663 normal eyes, 1616 keratoconus eyes, 210 keratoconus suspect eyes, 1519 myopic postoperative eyes, and 669 abnormal eyes). Each group was randomly divided into a training set (70% of the dataset) and a validation set (the remaining 30%). A multilayer perceptron network with a backpropagation learning algorithm was developed for the study. Indexes used to train the ANN were based on curvature and elevation of both the anterior and posterior corneal surfaces and the new corneal OCT indexes—based on corneal, stromal, and epithelial thicknesses.

**Results:**

For keratoconus detection, our ANN showed an accuracy of 98.6%, precision of 96%, recall of 97.9%, and F1-score of 96.9%. For keratoconus suspect detection, our ANN showed an accuracy of 98.5%, precision of 83.6%, recall of 69.7%, and F1-score of 76%.

**Conclusions:**

Compared to previous literature, the addition of new OCT-based epithelial and stromal thickness indexes improves ANN detection capacity of keratoconus suspect eyes. For already stablished keratoconus our ANN detection capacity is excellent, but equivalent to previous evidence without incorporating such new OCT-based indexes.

**Translational Relevance:**

OCT-based epithelial and stromal thickness indexes improve ANN detection capacity of keratoconus on its early stages.

## Introduction

Keratoconus is characterized by a progressive thinning, bulging, and distortion of the cornea, with secondary loss of vision caused by low- and high-order aberrations. Visual rehabilitation of advanced corneal ectasias requires penetrating or lamellar corneal transplantation techniques, which have several drawbacks, such as graft rejection, failure, and slow visual recovery because of high levels of induced postoperative astigmatism in relation with the suture and wound healing.^1^ For this reason, keratoconus is a major concern for refractive surgeons, because further weakening of the corneal tissue by laser surgery could trigger rapid worsening in subclinical keratoconic eyes. Thus it is essential to diagnose this disease early to avoid any corneal refractive surgery on these eyes and to possibly offer other treatment modalities, such as crosslinking, to halt its progression.^1^^,^^2^

Historically, the diagnosis of advanced keratoconus was made with the slit lamp by observing specific clinical signs such as the Fleischer ring and the Munson sign, but nowadays the diagnosis is made almost exclusively through corneal topographers and tomographs, which have made possible the diagnosis in early stages. However, the latter remains difficult in some cases and relies on the subjective interpretation from the clinician, so the development of a method for detecting this disease based on machine and deep learning methods is necessary for reliable early keratoconus detection. Deep learning is a branch of artificial intelligence in which layers of mathematical functions organized similar to the human cortex can be trained to recognize patterns in imaging and other data that are not immediately apparent, even to a skilled observer.

In ophthalmology, deep learning algorithms have proven to be effective in automating keratoconus diagnosis by using Placido based topographers.^3^^–^^6^ However, new combined Placido disc and high-resolution anterior segment optical coherence tomography (AS-OCT) topographers are currently available, and they provide new valuable data (such as reliable and reproducible corneal epithelial and stromal thicknesses maps) that could feed these neural networks to further enhance their keratoconus recognition capacity.^7^ In this sense, MS-39 (Costruzione Strumenti Oftalmici, Florence, Italy) uses a light source of 845 nm of wavelength, providing cross-sectional images with an axial resolution of 3.6 µm in an area of 16 mm. Accuracy of MS-39 outputs has been demonstrated in previous publications.^8^^–^^10^

The aim of the current study was to build a neural network using the information provided, in the form of derived indexes, by the MS-39 on a training dataset, to develop an automated keratoconus detection program and to assess its reliability for this purpose by using a validation dataset. The described classifier has already been implemented in the MS-39 keratoconus summary of Phoenix software since version 4.0.

## Material and Methods

### Patients and Setup

A cross-sectional study was conducted on a heterogeneous population from three different clinical settings: Vissum (Miranza group, Alicante, Spain), Magrabi Eye Hospital (Jeddah, Saudi Arabia), and Muscat Eye Laser Center (Muscat, Oman). The entire MS-39 topographer database from these three centers was used to extract anonymized patients’ data from daily practice.

The first examination of each patient (in case of multiple exams along time) was used, and only patients with at least three acquisitions per eye were included (routine practice in all study centers). Only one eye per included patient was randomly selected to adjust the effect of the correlation between fellow eyes on outcomes. Measurements with the best acquisition quality (in which the device provides a green-colored checkmark) were selected. Manual editing of the Placido rings or section was done when necessary (12% of the sample) to correct detection mistakes, such as irregularities or shadows from eyelashes and nose causing mistakes on the correct ring positioning or to increase the measurement coverage.

The three local institutional review boards approved this cross-sectional study, and there was no necessity to assign an ethics code because of the study's cross-sectional and anonymized design. The tenets of the Declaration of Helsinki were met in all stages of the study.

### Classification

**Figure 1.**
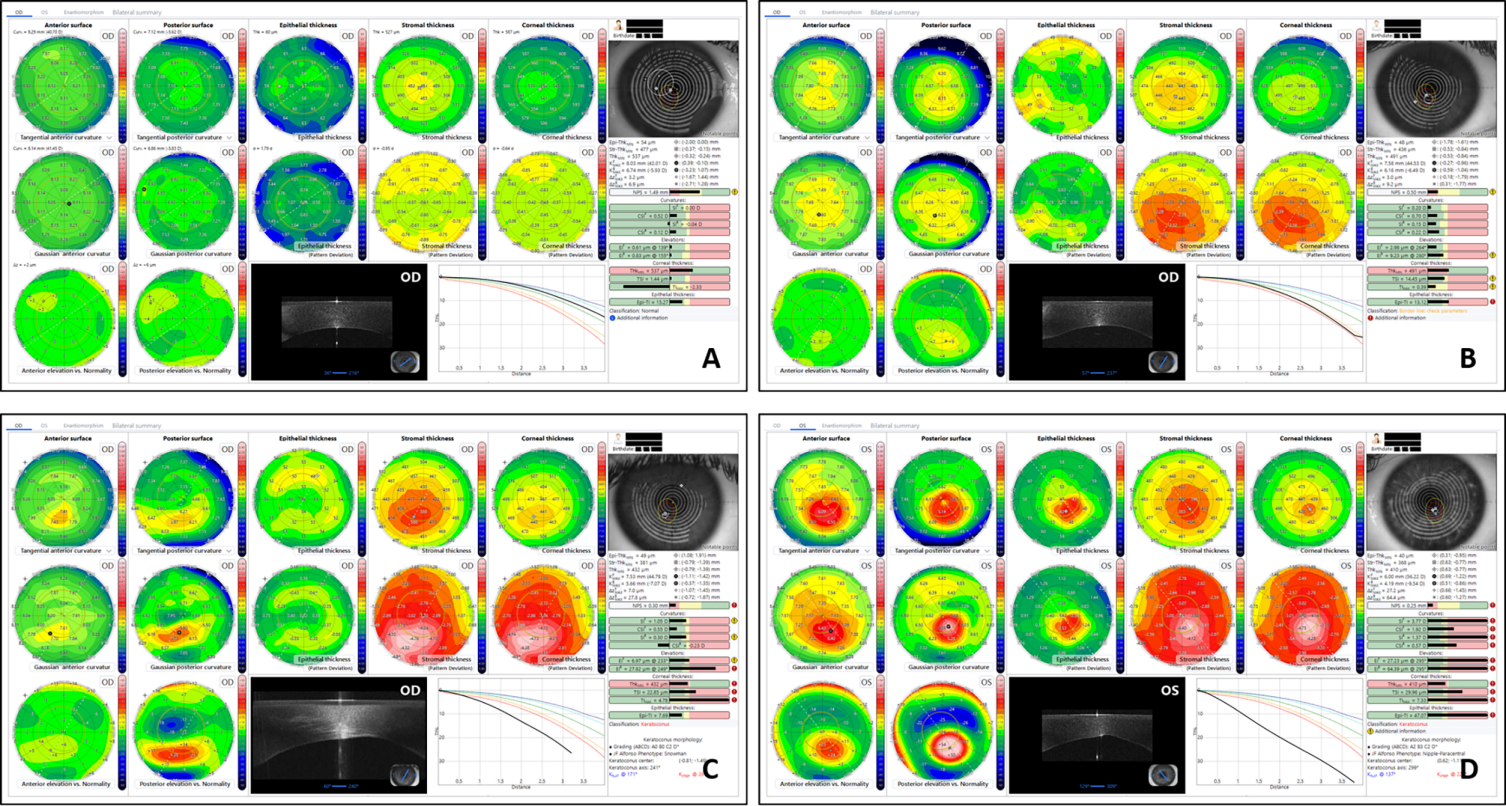
Example of a topographic pattern for a patient in the (**A**) normal subject group, (**B**) suspect keratoconus subject group, (**C**) keratoconus subject group (grade I according Amsler Krumeich or A0 B0 C2 according Belin classification), and (**D**) keratoconus subject group (grade II according Amsler Krumeich or A2 B3 C2 according Belin classification). The keratoconus summary uses curvature (anterior and posterior), elevation (anterior and posterior) and thickness (epithelial, stromal and corneal) data to facilitate the clinician in the disease diagnosis.

All selected topographies were classified in: normal subjects (N), patients with keratoconus (Kcn), patients with suspicious keratoconus (SKcn), and patients with previous myopic laser refractive surgery (Fig. 1). The remaining tomographs not considered as “normal” but also not fitting within any of the previous described groups were considered as “abnormal cornea” (Ab). *Keratoconus diagnosis* was based on the combination of the following data^7^: 3-mm I–S keratometric difference >1.4 D; central K >47.2 D; typical topographical map patterns for Kcn (round, oval, inferior steep, irregular, inferior-steep asymmetric bow tie, and symmetric or asymmetric bow tie with SRAX >21°); typical epithelial and stromal map patterns for Kcn (focal epithelial thinning over the corneal vertex with or without adjacent compensatory thickening, increased central epithelium/stroma ratio); central/paracentral or inferior focal steepening (anterior and/or posterior) and corneal thinning. We defined as *“keratoconus suspect”* all those cases where, after an overall and complete evaluation of the MS-39 analysis (anterior and posterior curvature and elevation, tomography, corneal aberrations, epithelial and stromal thicknesses, keratoconus indexes, enantiomorphism, rate of change of thickness/mm, etc.), it was not possible to establish the definitive diagnosis of keratoconus, but subtle or suspicious signs for it were present. Patients with subclinical keratoconus (topographic signs of keratoconus without clinical signs and best-corrected visual acuity of 20/20 or better) were considered as “keratoconus.”

As a first step, one independent observer (F.V.) selected all suitable high-quality topographies to be included in the study (one measurement per eye and one eye per patient). Then, three corneal and refractive surgery experts (J.A.B., A.E., J.A.) classified independently the whole database, a total of 6677 eyes, using the previous classification and according to their own expertise. Chosen classification among the three clinicians was not coincident in 770 out of the 6677 eyes (11.5%). For all such cases with at least one divergent opinion, the following protocol was applied:

• Subsequent meetings were performed to discuss these 770 eyes, seeking full agreement among the three clinicians.
• When full agreement was not achieved, the classification with the majority of votes was chosen.

The 6677 eyes were finally classified as follows (Fig. 1):

• 2663 normal eyes
• 1616 keratoconus eyes
• 210 keratoconus suspect eyes
• 1519 myopic post-op eyes
• 669 abnormal eyes

This classification was later used as a reference value for the trueness evaluation from the artificial neural network. From the 6677 eyes, 406 were finally excluded from statistical analysis because of scan quality deficiencies: 98 (1.47%) in the Normal group, 162 (2.43%) in the keratoconus group, five (0.07%) in the keratoconus suspect group, 56 (0.84%) in the myopic post-op group, and 85 (1.27%) in the abnormal group. Scan quality is automatically assessed by the device (quality indexes) by analyzing fixation deficiencies, coverage of keratoscopic images, corneal centration, tear film quality, coverage of the OCT scans, and more.

### Artificial Neural Network (ANN) Architecture

As previously performed,^3^^,^^11^^,^^12^ each study group was randomly divided into a training dataset (to be used for learning and to develop the keratoconus detection program) and a validation dataset (including the remaining eyes) to apply and test the resulting detection program.

The rule to choose the size of training and test datasets was to use a proportion of 70% for the training dataset and 30% for the test dataset. We shall take into account that the higher the training dataset, the better the classification model becomes, and more test datasets make the estimate error more accurate. Subsequently, the overall dataset was divided in a test dataset containing 770 normal subjects (28.9%), 436 (27%) keratoconus subjects, 66 (31.4%) keratoconus suspect subjects, 439 (28,9%) myopic postoperative subjects, and 176 abnormal subjects (26.3%). The training dataset contained the remaining cases.

A multilayer perceptron network with a backpropagation learning algorithm was developed for the study in which each network neuron is activated by means of a bipolar sigmoid activation function ranging in the interval [−1; 1]. The first layer (input layer) has a number of neurons equal to the number of indexes used for classification, and the last layer (output layer) has a number of neurons equal to the number of output class. The training set was preprocessed so that all input layers are normalized in the range [−1; 1] using an appropriate cutoff value; each output neuron corresponding to the winning class was set to one, whereas all other class neurons were set to zero. During the test phase, because the network response is not binary, the neuron with the highest output was marked as the certain one.

Because many parameters have deep influence on the network training process, a consistent number of training trials was carried out by modifying each time the learning rate, momentum, sigmoid activation function sigma, and number and size of hidden neurons layers. The network that provided fastest, steepest, and most stable error descent rate along with the best results on the test dataset was finally chosen and saved for further data analysis and results presentation. Table 1 summarizes the indexes used to train the ANN based on curvature and elevation of both the anterior and posterior corneal surfaces and the corneal and epithelial thickness data.

**Table 1.** Summary of Indexes Used to Train the ANN


SI^F^ and SI^B^—Symmetry index of the anterior and posterior surface	The symmetry index of the SI^F^ is defined as the difference of the mean anterior Gaussian curvature (expressed in diopters) of two circular zones centered in the inferior and superior hemispheres on the axis at 81°/261° or 99°/279° (according to the right or left laterality, respectively). Similarly, the symmetry index of the SI^B^ is defined based on posterior Gaussian curvature.
CSI^F^ and CSI^B^—Anterior and posterior center-surrounding index	The Center-Surrounding Index of the anterior curvature (CSI^F^) and the Center-Surrounding Index of the posterior curvature (CSI^B^) are defined as the difference of the mean Gaussian curvature expressed in diopters—anterior for CSI^F^ and posterior CSI^B^—of a central circular zone with a radius of 1.5 mm and the concentric annulus with a major radius of 3 mm and a minor radius of 1.5 mm.
EI^F^—Ectatic index anterior	The EI^F^ could be expressed as a multi-quadratic combination of Zernike coefficients of anterior surface decomposition such to maximize the difference between Normal and Keratoconus subjects as $$\begin{array}{c} \sum_{k=1}^{2}\sum_{n,m}\alpha_{nmk}{\left(c_{n}^{m}\right)}^{k} \end{array}$$ where $c_{n}^{m}$ is the Zernike coefficient with radial order n and azimuthal frequency *m*, α_*nmk*_ is its relative weight.
EI^B^—Ectatic index posterior	The EI^B^ could be expressed as a multi-quadratic combination of Zernike coefficients of posterior surface decomposition such to maximize the difference between normal and keratoconus subjects as $$\begin{array}{c} \sum_{k=1}^{2}\sum_{n,m}\alpha_{nmk}{\left(c_{n}^{m}\right)}^{k} \end{array}$$ where $c_{n}^{m}$ is the Zernike coefficient with radial order n and azimuthal frequency *m*, α_*nmk*_ is its relative weight.
RMS^F^ and RMS^B^—Root mean square of the anterior and posterior surface	RMS^F^ represents the deviation of the anterior elevation from a reference surface, with physiological asphericity Q = −0.2 (average value of healthy normal population) and best-fit apical radius and toricity calculated on an 8 mm diameter circular zone centered on corneal vertex. Similarly RMS^B^ is calculated in reference to an aspho-toric surface, with physiological asphericity Q = −0.3.
Thk_Min_—Minimum corneal thickness	The minimum corneal thickness is in the central 8 mm diameter zone.
SI^THK^—Symmetry index of the corneal thickness	The symmetry index of the thickness is defined as the difference of the mean thickness of two circular zones centered in the inferior and superior hemispheres having their center on the axis at 81°/261° or 99°/279° (according to the right or left laterality, respectively).
%TI—Max percentage thickness increase	%TI index is defined as the maximum difference of percentage thickness increase of current pachymetry and the 95th percentile of the normal healthy population.
%EpiTI—Max percentage of epithelial thickness increase	Similar to %TI, %EpiTI index is defined as the maximum difference of Percentage of Epithelial Thickness Increase between of current pachymetry and the 95th percentile of normal healthy population.
K_Max_^F^ and K_Max_^B^—Anterior and posterior apical value	K_Max_^F^ is defined as the maximum value of the anterior gaussian curvature map. Similarly K_Max_^B^ is defined as the maximum value of the posterior gaussian curvature map.
K_avg_^F^—Average anterior curvature	If we consider the values of average axial curvatures (in mm) of the two principal meridians of the front corneal surface *Rf_f_* and *Rf_s_*. K_avg_^F^ is calculated as their average. Both *Rf_f_* and *Rf_s_* are, for their respective meridian, the average values in the central zone within a diameter of 3 mm.
K_avg_^B^—Average posterior curvature	If we consider the values of average axial curvatures (in mm) of the two principal meridians of the posterior corneal surface *Rb_f_* and *Rb_s_*. K_avg_^B^ is calculated as their average. Both *Rb_f_* and *Rb_s_* are, for their respective meridian, the average values in the central zone within a diameter of 3 mm.
DZ_Max_^F^ and DZ_Max_^B^—Most elevated value of elevation anterior and posterior map	DZ_Max_^F^ is defined as the maximum value of the deviation of the anterior elevation from a reference surface with physiological asphericity Q = −0.2 and best-fit apical radius and toricity calculated on an 8 mm diameter circular zone centered on corneal vertex. Similarly DZ_Max_^B^ is calculated in reference to an aspho-toric surface, with physiological asphericity Q = −0.3.
Spread of the NPS	According to the following points, we can define the NPS as the average distance of the notable points from their barycenter: • $PEpiThk_{Min}$. the location of the minimum of epithelial thickness layer. • $PStrThk_{Min}$. the location of the minimum of stromal thickness layer. • $PThk_{Min}$. the location of the minimum of corneal thickness. • $PK_{max}^{F}$ the location of maximum value in diopters (or the minimum in mm) of the Gaussian curvature for the anterior corneal surface. • $PK_{max}^{F}$ the location of maximum value of the Gaussian curvature for the posterior corneal surface.• P DZ_Max_^F^ the location of the maximum height derived from the anterior elevation vs. normality map. • P DZ_Max_^B^ the location of the maximum height derived from the posterior elevation versus normality map.

NPS, notable points radius; SI^B^, posterior curvature; SI^F^, anterior curvature.

Descriptive statistics were conducted for each sub-class, followed by a one-sample Kolmogorov-Smirnov test (*P* > 0.05) and receiver operating characteristic (ROC) curve analysis applied to each derived parameter. All calculations were executed using the statistical software IBM SPSS Statistics (version-27).

## Results

### Demographic Analysis

Overall 6677 eyes (3346 OD and 3331 OS) from an equal number of patients (one eye per patient) were enrolled. The enrolled subjects had the following distribution by ethnicity:

• 2206 Arabic subjects (33%)
• 1615 Caucasian subjects (24.2%)
• 1206 Black Indian subjects (18.1%)
• 946 Asian subjects (14.2%)
• 704 Black African subjects (10.5%)

A descriptive statistic of gender and age is reported in Supplemental Table S1. The distribution of the target spherical correction for the subjects in the myopic postoperative eyes group is shown in Supplemental Table S2. Supplemental Table S3 shows the distribution for patients in KC group using the Amsler-Krumeich grading system.

### Descriptive Statistics of Indexes

In Supplemental Table S4 we present a descriptive statistics for each of the endpoints that fed the ANN. We performed a Kolmogrov-Smirnov test stratified by group to assess the normality of the distribution of these indexes. Because the vast majority of reported indexes resulted in not normally distributed statistics (*P* < 0.05; bold numbers), we decided to provide a description of each endpoint in each class by percentiles and histograms (Supplemental Table S5). The normality range for a subject arises from the following statistic: first and ninety-ninth percentiles are considered the abnormality thresholds, and the values from the first and fifth percentiles and ninety-fifth and ninety-ninth percentiles are considered borderline.

### Sensitivity and Specificity (Normal Versus Keratoconus Groups and Normal Versus Keratoconus Suspect Groups)

We studied the closeness of agreement between the average value obtained from a large series of test results and an accepted reference value. In the current study the latter was defined as the classification performed by the three expert clinicians involved in the study.

**Figure 2.**
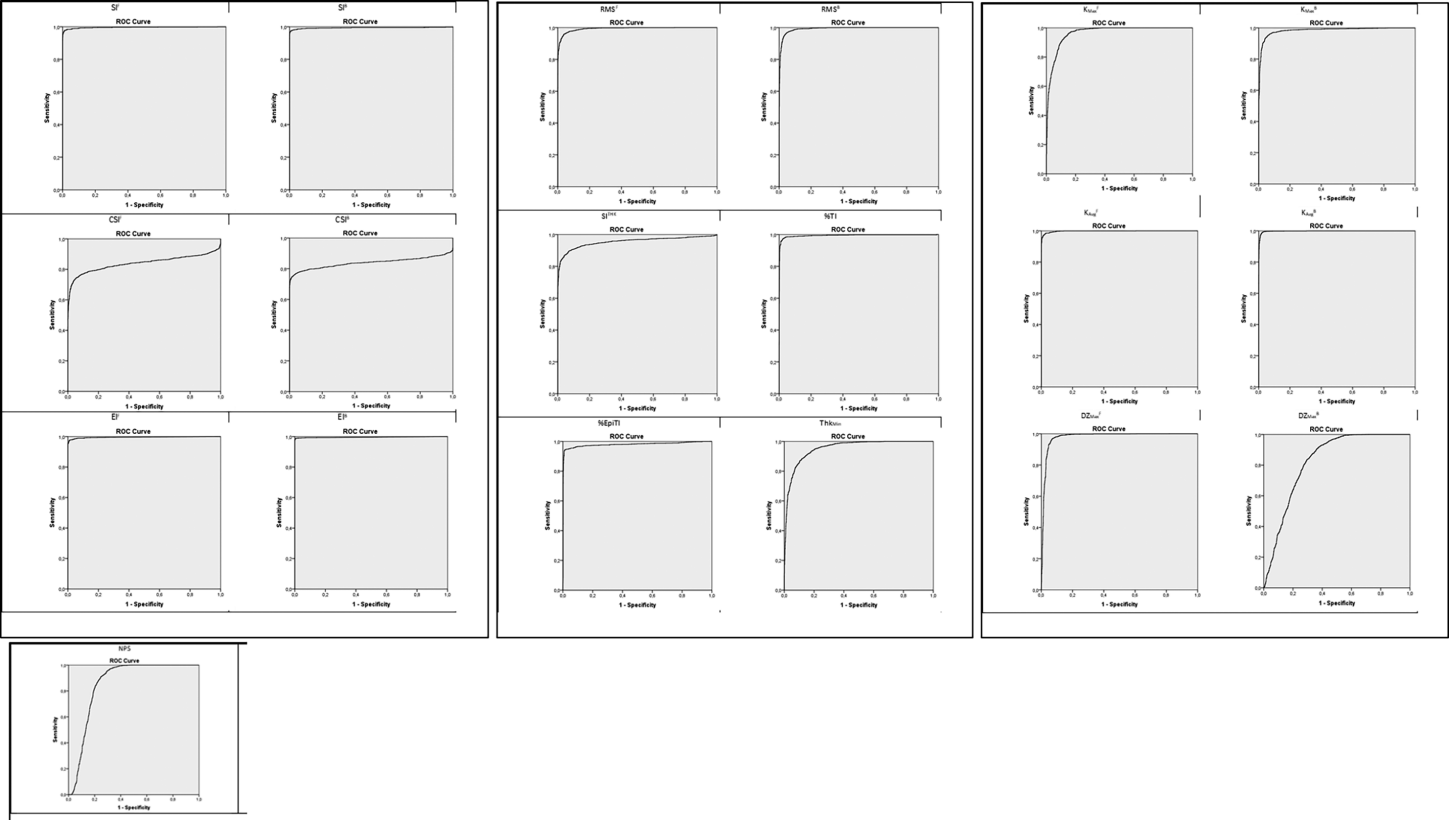
ROC curve for specificity and sensitivity for SI^F^, SI^B^, CSI^F^, CSI^B^, EI^F^, EI^B^, RMS^F^, RMS^B^, SI^THK^, %TI, %EpiTI, Thk_Min_, K_Max_^F^, K_Max_^B^, K_Avg_^F^, K_Avg_^B^, DZ_Max_^F^, DZ_Max_^B^, and NPS parameters (from top-left to bottom-right) for normal and keratoconus subject groups.

**Figure 3.**
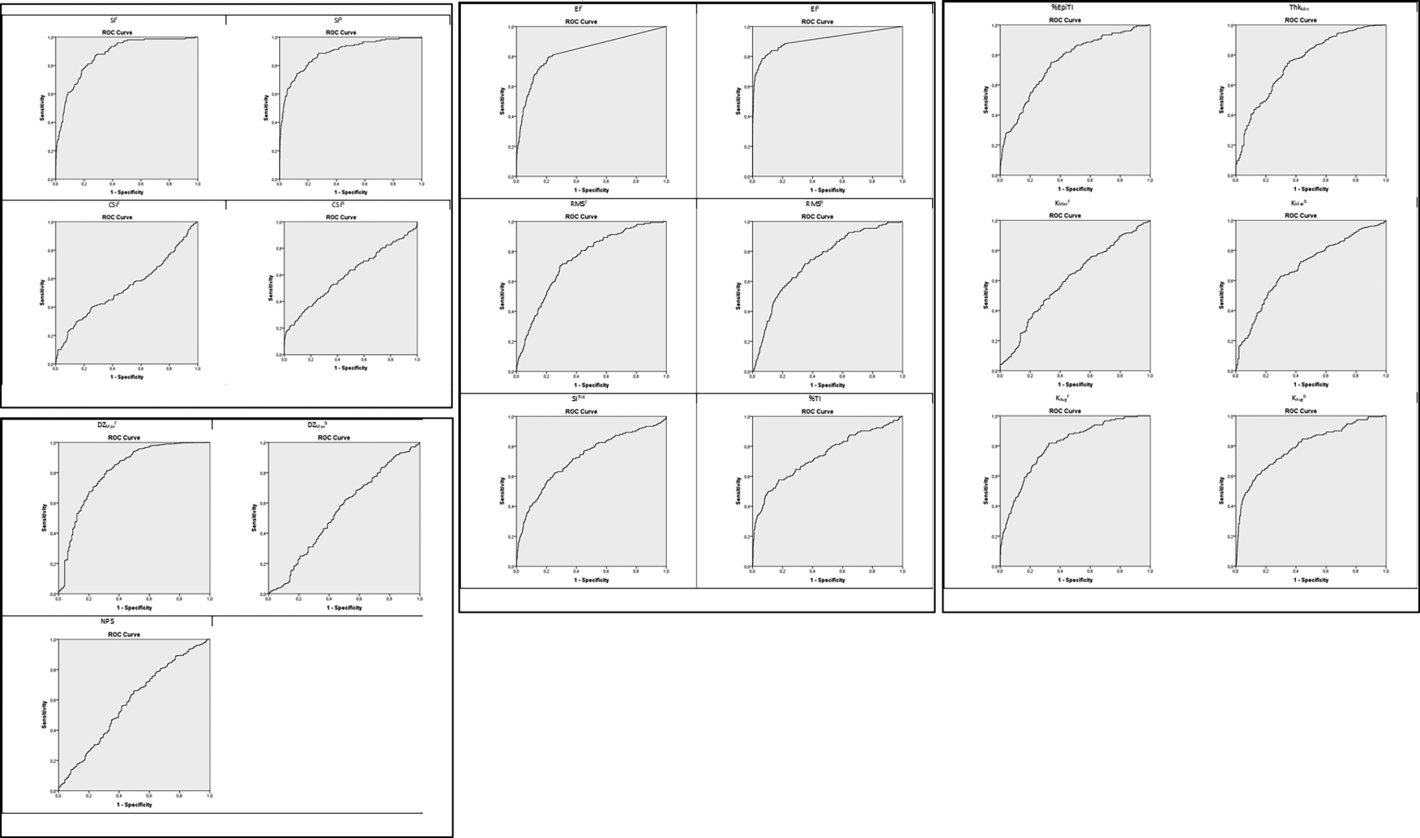
ROC curve for specificity and sensitivity for SI^F^, SI^B^, CSI^F^, CSI^B^, EI^F^, EI^B^, RMS^F^, RMS^B^, SI^THK^, %TI, %EpiTI, Thk_Min_, K_Max_^F^, K_Max_^B^, K_Avg_^F^, K_Avg_^B^, DZ_Max_^F^, DZ_Max_^B^, and NPS parameters (from top-left to bottom-right) for normal and suspect keratoconus subject groups.

In Table 2 and Figures 2 and 3 we show information about the performance of each single index in the discrimination of normal subjects versus keratoconus subjects class, and normal subjects versus keratoconus suspect subjects class. For the sake of simplicity, we decided to omit abnormal and myopic postoperative classes because the main aim of the study was the detection of keratoconus and keratoconus suspect classes. Hosmer et al.^13^ described a general guideline for the evaluation of the area under the ROC curve parameter:

• 0.5 = No discrimination
• 0.5–0.7 = Poor discrimination
• 0.7–0.8 = Acceptable discrimination
• 0.8–0.95 = Excellent discrimination
• >0.95 = Outstanding discrimination

**Table 2.** Area Under the ROC Curve and Discrimination Capacity

	(N Vs Kcn)		(N Vs sKcn)	
	Area Under the ROC	Discrimination	Area Under the ROC	Discrimination
SI^F^	0.996	Outstanding	0.886	Excellent
SI^B^	0.993	Outstanding	0.537	Poor
CSI^F^	0.840	Excellent	0.608	Poor
CSI^B^	0.839	Excellent	0.826	Excellent
EI^F^	0.995	Outstanding	0.908	Excellent
EI^B^	0.996	Outstanding	0.745	Acceptable
RMS^F^	0.993	Outstanding	0.736	Acceptable
RMS^B^	0.992	Outstanding	0.659	Poor
SI^THK^	0.955	Outstanding	0.717	Acceptable
%TI	0.993	Outstanding	0.736	Acceptable
%EpiTI	0.951	Outstanding	0.749	Acceptable
Thk_Min_	0.954	Outstanding	0.750	Acceptable
K_Max_^F^	0.964	Outstanding	0.611	Poor
K_Max_^B^	0.984	Outstanding	0.697	Poor
DZ_Max_^F^	0.998	Outstanding	0.802	Excellent
DZ_Max_^B^	0.998	Outstanding	0.795	Acceptable
NPtsR	0.979	Outstanding	0.808	Excellent
K_Avg_^F^	0.824	Excellent	0.550	Poor
K_Avg_^B^	0.858	Excellent	0.886	Excellent

Test dataset analysis reveals that the chosen indexes are able to discriminate well between normal subjects and keratoconus subjects. It also reveals that when a clear topographical diagnosis does not discriminate between the two classes (i.e., in Keratoconus Suspect eyes) an objective discrimination based on a single index becomes difficult (Table 2 and Fig. 3).

### Accuracy, Precision, and Recall

Table 3 shows the results with reference to the implemented ANN classifier.^14^^,^^15^ The accuracy is a measure of generic ability of the network to correctly classify a new case. The accuracy of our ANN classifier was 98.6% for keratoconus and 98.5% for keratoconus suspects.

**Table 3.** Confusion Matrix of the Classification of the Test Dataset and Performance of the Classifier

	Classification				
Class	Abn	Kcn	MyPO	Normal	SKcn
Abnormal	144 (81.82%)	9 (5.11%)	8 (4.55%)	14 (7.95%)	1 (0.57%)
Keratoconus	4 (0.92%)	427 (97.94%)	1 (0.23%)	0 (0.00%)	4 (0.92%)
Myopic postop	7 (1.59%)	0 (0.00%)	427 (97.27%)	5 (1.14%)	0 (0.00%)
Normal	1 (0.13%)	0 (0.00%)	6 (0.78%)	759 (98.57%)	4 (0.52%)
Suspect Kcn	4 (6.06%)	9 (13.64%)	0 (0.00%)	7 (10.61%)	46 (69.70%)
True-positive	144	427	427	759	46
False-positive	16	18	15	26	9
False-negative	32	9	12	11	20
True-negative	1695	1433	1433	1091	1812
Accuracy	97.46%	98.57%	98.57%	98.04%	98.46%
Precision	90.00%	95.96%	96.61%	96.69%	83.64%
Recall	81.82%	97.94%	97.27%	98.57%	69.70%
F1	85.71%	96.94%	96.94%	97.62%	76.03%

MyPO, myopic laser refractive surgery.

The precision is a measure of the specificity (capability of the classifier of not classifying false positives; positive predicative value). The precision of our ANN classifier was 96% for keratoconus and 83.6% for keratoconus suspects.

The recall is a measure of the sensitivity of the classifier (capability of the classifier of classifying true positives). The recall of our ANN classifier was 97.9% for keratoconus and 69.7% for keratoconus suspects.

F1-score is a measure that combines precision and recall (it is the harmonic mean of precision and recall). The F1 of our ANN classifier was 96.9% for keratoconus and 76% for keratoconus suspects.

## Discussion

Thanks to modern corneal topography, established keratoconus diagnosis has become straightforward. However, such diagnosis in its earliest stages remains challenging and relies mostly on clinicians’ experience and intuition. Diagnosis and the importance of detecting those suspicious cases with possible early ectatic disease in laser vision correction (not always performed by skilled cornea experts) makes it essential to support clinicians with automatic methods for detecting keratoconus based on machine and deep learning.

Keratoconus grading classification systems went through many stages of improvement and development prompted by the progress in diagnostic devices at a given time.^10^^–^^12^ However, one or the other or a combination of systems have not yet been established as the reference of care for clinician and surgeon use in real practice. Because of this, the goal of our machine learning and in general for the majority of keratoconus neural networks is not to classify a keratoconus case based on a specific degree of severity according to a given classification but to identify the disease in the simpler form of a dichotomy (yes/no) but with the maximal sensitivity and specificity possible. By splitting keratoconus cases from those with suspicious signs but without enough evidence to establish the definitive diagnosis of the disease, we tried to highlight those eyes in which a close topographic follow-up is recommended and mainly reconsider any form of corneal laser refractive surgery. In this context, modern topographers, by adding high-resolution AS-OCT to their technology, are able to provide repeatable and reliable corneal, epithelial, and stromal maps, and the variables extracted from them have proven good diagnostic value in the detection of keratoconus^10^ and in forme fruste and subclinical keratoconus forms.^7^ Thus, considering that previously reported neural networks^3^^,^^5^^,^^6^ used predominately keratometric and tomographic data, the addition of these new reliable data from combined Placido disc and high-resolution AS-OCT topographers could improve the detection capacity of these classification systems, as we did in the current study. All indexes used to train our ANN (Table 1) showed “excellent” or “outstanding” keratoconus discrimination capacity according to their area under the ROC (Table 2). In consequence, our neural network performed very well on identifying keratoconus cases, with an accuracy of 98.6%, a precision (specificity) of 96%, a recall (sensitivity) of 97.9%, and an F1 of 96.9% (Table 3). If we pay closer attention to the recall performance of our ANN, in Table 3 we can see that 427 of the 436 keratoconus eyes (97.9%) were correctly identified by the ANN. Four mistaken eyes were classified as “abnormal”: these cases showed a very advanced keratoconus with abnormal epithelial thickness and corneal scars, leading to the misclassification. The other four mistaken eyes were classified by the ANN as “keratoconus suspect.” In any case, all eight mistaken eyes were classified with a warning to the clinician, with only one eye (0.2%) remaining that was totally mistaken by the ANN because it was classified as myopic post-op. No keratoconus eye was incorrectly classified as normal.

 “Forme fruste keratoconus,” “subclinical keratoconus,” and “keratoconus suspect” are terms that are frequently mixed and used indistinctly by many corneal and refractive surgeons, creating confusion. In theory, the term “forme fruste keratoconus” applies to clinically and topographically normal fellow eyes of patients with manifest KC in the other eye,^7^ but these eyes don't really present topographic signs of disease, and so we did not consider them for our ANN as “suspects”. Moreover, the concept of “subclinical keratoconus” actually applies to forms of real disease but in its early stages (without visual involvement). For this reason, we decided to include those eyes with “subclinical keratoconus” within the “keratoconus” group (because they are obvious cases of disease) and to use for our ANN classifier only those eyes with a suspicious disease, with the term “keratoconus suspect” applied to all those cases without enough topographic signs to establish the definitive diagnosis of keratoconus but showing subtle and suspicious signs for it. For such “keratoconus suspect” cases, the indexes used to train our ANN lost discrimination capacity (according to their area under the ROC), but the majority of them kept it at least on an “acceptable” or even an “excellent” degree (Table 2). Thus, although our ANN performed worse on identifying such “keratoconus suspects,” it still did relatively well, demonstrating an accuracy of 98.46%, a precision (specificity) of 83.6%, a recall (sensitivity) of 69.7%, and an F1 of 76% (Table 3). Again, if we analyze further the recall performance of our ANN for this group (Table 3), we can see that 46 of the 66 keratoconus suspect eyes (69.7%) were correctly identified by the ANN. Nine eyes were mistakenly classified as “keratoconus,” and the other four mistaken eyes were classified as “abnormal” by the ANN, which meant that only seven eyes (10.6%) were the real mistake performed by the ANN because they were incorrectly classified as “normal.” The unavoidable relative subjectivity on the definition used for this group of suspects (suspicious topographic signs without objective keratoconus disease) made this diagnosis more dependent on each evaluator’s criteria, and this limitation probably justifies the lower performance of the ANN for this group of suspects compared to the keratoconus group. Other limitations of the study may include the following:

• Although the primary objective is the early detection of keratoconus, the ANN does not categorize the progression state of the disease. Although the indexes used in this study may have the potential for such application, we deem this task to be beyond the scope of the current research.
• As outlined earlier, the described ANN underwent evaluation using an extensive and diverse validation set sourced from three distinct centers and exhibiting commendable multiethnic representation. However, we did not use an external dataset for definitive validation.

In a previous study, a similar methodology was applied using a Scheimpflug camera combined with Placido disc topographer (Sirius; Costruzione Strumenti Oftalmici).^3^ By using only anterior corneal surface data, they reported an accuracy of 96.9% and a precision of 94.6%. However, by including anterior and posterior corneal surfaces as well as pachymetric data, accuracy and precision improved to 98.2% and 97.9% respectively. These results are similar to the outcomes obtained with our ANN in the current study where we also included new variables such as all epithelial thickness indexes (accuracy of 98.6%, precision of 96%). On the other hand, in this same previous study with Sirius topographer,^3^ they also reported an accuracy and precision of 97.3% and 78.8%, respectively, for subclinical keratoconus detection. However, this group included “subclinical”, “forme fruste” and “keratoconus suspects” cases, whereas in our ANN we decided to be more strict and include only true “keratoconus suspect” cases (excluding eyes with forme fruste and subclinical forms) as already explained. Nevertheless, despite our more strict criteria, we got better outcomes, with an accuracy of 98.5% and a precision of 83.6% for this group, highlighting the conclusion that the inclusion of all epithelial indexes to the ANN improves its detection capacity for true “keratoconus suspect” cases (those considered almost normal but with some subtle signs of disease), which actually are the ones that present the real challenge in clinical practice. In contrast, the inclusion of these new variables to the ANN does not seem to improve its performance for the detection of established keratoconus, where the baseline detection capacity is already outstanding.

Similar studies have also been published with alternative platforms. Using Pentacam (Oculus, Wetzlar, Germany) and indexes from both the anterior and posterior cornea to feed the ANN, Ghaderi et al.^5^ reported a global accuracy (normal vs. keratoconus cases) of 98.2%. Subclinical, forme fruste and suspicious cases were not evaluated. Using this same platform, Lopes et al.^16^ described the “Pentacam random forest index” (PRFI) using a multicenter database. Such PRFI obtained a 100% sensitivity for clinical ectasia in their sample, performing statistically better than the Belin/Ambrósio display index. They did not consider keratoconus suspects as we did in our study, but they analyzed the performance of their PRFI in a sample of forme fruste keratoconus, describing a sensitivity of 85.2%. A recent study by Ambrosio et al.^17^ optimized this ANN by including biomechanical indexes obtained with Corvis ST (Oculus; tomographic-biomechanical index). For clinical ectasia they described a 98.7% sensitivity and 99.2% specificity, and for detecting forme fruste keratoconus a 84.4% sensitivity and 90.1% specificity. They concluded that ANN optimization to integrate Scheimpflug-based corneal tomography and biomechanical assessments augments accuracy for ectasia detection. Using Orbscan raw data on a convolutional neural network, Zeboulon et al.^18^ reported an accuracy of 99.3%. In line with our study, Shi et al.^4^ reported on the relevance of including corneal epithelial indexes obtained from high-resolution OCT devices to increase the detection capacity of ANNs in subclinical keratoconus. In this study, they used two separate devices (Pentacam and a high-resolution OCT prototype system), and they got 99% precision for keratoconus eyes and 93% precision for subclinical keratoconus eyes. They also observed that ANN discrimination capacity for keratoconus eyes was excellent and equal regardless of whether the information came from the Pentacam only, AS-OCT only, or a combination of both devices. Thus, adding the information from AS-OCT did not make the ANN better for keratoconus eyes detection, as we observed in the current study. However, for subclinical keratoconus diagnosis, the discrimination capacity significantly improved when both devices were combined in comparison to each of them separately, thus sharing our same conclusion: the addition of epithelial indexes from high-resolution AS-OCT devices increases the discrimination capacity of ANNs on the diagnosis of subclinical or suspicious keratoconus eyes. Nevertheless, we shall take into account that their study sample was limited (38 keratoconus, 33 subclinical, and 50 normal eyes, compared to the 1616, 210, and 2663 eyes, respectively, in our study). So, to the best of our knowledge, we are providing the first ANN using both AS-OCT and Placido disc topographer data with the largest training and validation datasets, and with a device (MS39) that is commercially available and already combines both technologies within the same device and with only one capture, which involves a clear advantage on daily practice.

In conclusion, ANNs are essential tools to aid ophthalmologists in keratoconus screening. The addition of the newer epithelial and stromal thickness indexes from high-resolution AS-OCT technology does improve their detection capacity of the earliest forms of disease (keratoconus suspects), although it does not improve their performance for already stablished keratoconus (being already outstanding). Topographers combining all necessary technology are already commercially available (such as the MS39 used in the current study), providing such automatic screening with only one measurement. Finally, according to recent evidence,^17^ early keratoconus detection capacity of these new OCT topography based ANNs could potentially be further enhanced by including biomechanical indexes, and further studies may focus on this regard. We shall remember that all automatic screening systems should be applied as an additional tool and not in substitution to other clinical data.

## Supplementary Material

Supplement 1

Supplement 2

Supplement 3

Supplement 4

Supplement 5
