# Supplementary material for: Artificial Neural Network for Automated Keratoconus Detection Using a Combined Placido Disc and Anterior Segment Ocular Coherence Tomography Topographer
Source: Transl Vis Sci Technol. 2024 Apr 8;13(4):13. doi: 10.1167/tvst.13.4.13 (PMC11005070; doi:10.1167/tvst.13.4.13)
Supplement: Supplement 1 [file tvst-13-4-13_s001.pdf]

Gender and age by group	Male	Female	Min Age	50th perc.	Max Age	Avg Age	SD
Normal	1324	1339	18	29	92	33.9	14.8
Suspect keratoconus	108	102	18	29	74	32.2	13.5
Keratoconus	801	815	18	29	75	31.1	10.7
Myopic Post op	756	763	18	31	87	32.9	10.2
Abnormal	330	339	18	48	89	48.6	17.4

Supplemental Table 1. Descriptive statistics by gender and age for the Normal, Suspect keratoconus, Keratoconus, Myopic post-op and Abnormal groups.
