# Supplementary material for: Artificial Neural Network for Automated Keratoconus Detection Using a Combined Placido Disc and Anterior Segment Ocular Coherence Tomography Topographer
Source: Transl Vis Sci Technol. 2024 Apr 8;13(4):13. doi: 10.1167/tvst.13.4.13 (PMC11005070; doi:10.1167/tvst.13.4.13)
Supplement: Supplement 2 [file tvst-13-4-13_s002.pdf]

Target spherical equivalent correction [D]	Number of subjects
**<-6.5**	90 (5.9%)
**[-6.5, -5.5)**	90 (5.9%)
**[-5.5, -4.5)**	142 (9.3%)
**[-4.5, -3.5)**	231 (15.2%)
**[-3.5, -2.5)**	289 (19.0%)
**[-2.5, -1.5)**	306 (20.1%)
**[-1.5, -0.5)**	271 (24.4%)

Supplemental Table 2. Descriptive statistics by target spherical equivalent correction in myopic post-op group.
