# Supplementary material for: Artificial Neural Network for Automated Keratoconus Detection Using a Combined Placido Disc and Anterior Segment Ocular Coherence Tomography Topographer
Source: Transl Vis Sci Technol. 2024 Apr 8;13(4):13. doi: 10.1167/tvst.13.4.13 (PMC11005070; doi:10.1167/tvst.13.4.13)
Supplement: Supplement 3 [file tvst-13-4-13_s003.pdf]

Amsler-Krumeich grading	Number of subjects
**Stage I**	374 (23.1%)
**Stage II**	526 (32.5%)
**Stage III**	395 (24.4%)
**Stage IV**	321 (19.9%)

Supplemental Table 3. Descriptive statistics by stage in Keratoconus group.
