# Supplementary material for: Artificial Neural Network for Automated Keratoconus Detection Using a Combined Placido Disc and Anterior Segment Ocular Coherence Tomography Topographer
Source: Transl Vis Sci Technol. 2024 Apr 8;13(4):13. doi: 10.1167/tvst.13.4.13 (PMC11005070; doi:10.1167/tvst.13.4.13)
Supplement: Supplement 4 [file tvst-13-4-13_s004.pdf]

One-Sample Kolmogorov-Smirnov Test										
	Test Stat.	Asymp. Sig.	Test Stat.	Asymp. Sig.	Test Stat.	Asymp. Sig.	Test Stat.	Asymp. Sig.	Test Stat.	Asymp. Sig.
	**Normal**		**Suspect K**		**Keratoconus**		**Myopic post-op**		**Abnormal**	
**SI^F**	0.118	0.000	0.020	0.017	0.119	0.000	0.086	0.000	0.140	0.000
**SI^B**	0.081	0.018	0.019	0.027	0.104	0.000	0.073	0.000	0.137	0.000
**CSI^F**	0.094	0.003	0.055	0.000	0.181	0.000	0.077	0.000	0.149	0.000
**CSI^B**	0.123	0.000	0.034	0.000	0.138	0.000	0.060	0.000	0.095	0.000
**EI^F**	0.189	0.000	0.403	0.000	0.113	0.000	0.371	0.000	0.315	0.000
**EI^B**	0.131	0.000	0.427	0.000	0.100	0.000	0.328	0.000	0.315	0.000
**RMS^F**	0.115	0.000	0.101	0.000	0.129	0.000	0.128	0.000	0.145	0.000
**RMS^B**	0.074	0.042	0.136	0.000	0.138	0.000	0.306	0.000	0.290	0.000
**Thk_{MIN}**	0.040	**0.200***	0.018	**0.065***	0.062	0.000	0.018	**0.200***	0.081	0.000
**SI^{Thk}**	0.057	**0.200***	0.020	0.016	0.087	0.000	0.053	0.000	0.147	0.000
**%TI**	0.109	0.000	0.124	0.000	0.076	0.000	0.023	**0.058***	0.059	0.000
**%EpiTI**	0.369	0.000	0.419	0.000	0.380	0.000	0.423	0.000	0.240	0.000
**$K_{Max}{}^F$**	0.045	**0.200***	0.020	0.023	0.076	0.000	0.032	0.001	0.078	0.000
**$K_{Max}{}^B$**	0.081	0.017	0.081	0.000	0.051	0.000	0.104	0.000	0.037	**0.056***
**$K_{avg}{}^F$**	0.055	**0.200***	0.014	**0.200***	0.108	0.000	0.062	0.000	0.125	0.000
**$K_{avg}{}^B$**	0.053	**0.200***	0.025	0.001	0.275	0.000	0.298	0.000	0.361	0.000
**$Dz_{Max}{}^F$**	0.133	0.000	0.062	0.000	0.126	0.000	0.126	0.000	0.132	0.000
**$Dz_{Max}{}^B$**	0.099	0.001	0.113	0.000	0.119	0.000	0.277	0.000	0.140	0.000
**NPtsR**	0.108	0.000	0.034	0.000	0.197	0.000	0.039	0.000	0.026	0.200

Supplemental Table 4. Kolmogorov-Smirnov test on each endpoint for each group to assess the normality of the distribution of these indices (significance < 0.05; bold numbers*).
