# Supplementary material for: Artificial Neural Network for Automated Keratoconus Detection Using a Combined Placido Disc and Anterior Segment Ocular Coherence Tomography Topographer
Source: Transl Vis Sci Technol. 2024 Apr 8;13(4):13. doi: 10.1167/tvst.13.4.13 (PMC11005070; doi:10.1167/tvst.13.4.13)
Supplement: Supplement 5 [file tvst-13-4-13_s005.pdf]

	Mean	SD	Minimum	Maximum	Percentiles				
					1	5	50	95	99
**Normal**									
**SI^F**	0.1572	0.3509	-1.14	1.48	-0.67	-0.43	0.15	0.76	1.02
**SI^B**	0.0307	0.0791	-0.29	0.41	-0.15	-0.10	0.03	0.16	0.24
**CSI^F**	0.5267	0.4629	-1.07	3.46	-0.51	-0.17	0.51	1.28	1.98
**CSI^B**	0.1570	0.0952	-0.14	0.50	-0.05	0.01	0.15	0.32	0.40
**EI^F**	0.1185	0.3010	0	2.35	0	0	0	0.76	1.49
**EI^B**	0.0737	0.2017	0	1.95	0	0	0	0.50	0.96
**RMS^F**	2.8527	1.1418	0.82	12.21	1.18	1.48	2.61	5.06	6.75
**RMS^B**	5.4146	2.6068	1.63	34.86	2.34	2.82	4.85	9.80	15.29
**Thk_{MIN}**	541.0	33.8	426	695	464	487	541	596	625
**SI^{Thk}**	5.5547	3.6937	-11.44	19.84	-2.94	-0.04	5.45	11.92	14.85
**%TI**	-1.0487	1.1077	-9.79	7.96	-6.49	-2.15	-1.04	0.39	1.49
**%EpiTI**	-0.9332	0.1860	-1.11	1.00	-1.05	-1.01	-0.96	-0.83	-0.03
**$K_{Max}{}^F$**	7.5219	0.2914	6.62	8.68	6.83	7.04	7.53	7.99	8.15
**$K_{Max}{}^B$**	6.1694	0.3750	1.56	7.15	4.95	5.63	6.20	6.66	6.90
**$K_{avg}{}^F$**	7.7148	0.2722	6.77	8.88	7.11	7.26	7.72	8.16	8.35
**$K_{avg}{}^B$**	6.4810	0.2895	5.65	8.10	5.84	6.02	6.46	6.98	7.27
**$Dz_{Max}{}^F$**	4.5913	1.7345	1.00	14.96	1.68	2.27	4.34	7.91	10.18
**$Dz_{Max}{}^B$**	8.7044	3.8918	2.33	55.86	3.46	4.47	8.01	14.78	23.53
**NPtsR**	1.4554	0.4835	0.18	2.89	0.51	0.70	1.44	2.27	2.58
**Keratoconus**									
**SI^F**	5.4639	4.1897	-1.48	39.90	0.60	1.23	4.44	13.03	20.13
**SI^B**	1.4949	1.0346	-0.46	8.08	0.12	0.34	1.25	3.39	5.73
**CSI^F**	4.4408	5.7769	-2.73	52.61	-1.74	-0.65	2.84	13.83	29.70
**CSI^B**	1.2580	1.4231	-1.59	9.62	-0.52	-0.20	0.86	3.98	7.23
**EI^F**	11.0995	8.3325	0	71.27	0.41	2.08	9.17	27.18	39.96
**EI^B**	12.7910	8.2327	0	57.70	0.65	2.84	10.89	28.63	39.32
**RMS^F**	17.8659	12.5170	2.13	101.88	3.84	5.43	14.68	40.60	68.68
**RMS^B**	34.4133	24.4962	5.36	286.00	7.84	10.80	28.43	76.65	130.02
**Thk_{MIN}**	442.2	57.2	160	584	241	343	449	519	549
**SI^{Thk}**	26.5728	15.8175	-61.59	117.35	-0.44	7.40	23.61	55.35	82.37
**%TI**	6.5288	4.0338	-5.78	23.79	-0.32	1.32	5.81	13.70	19.98
**%EpiTI**	0.8387	0.4505	-1.04	1.00	-0.97	-0.75	0.99	1.00	1.00
**$K_{Max}{}^F$**	6.2809	0.7871	3.13	7.85	3.79	4.65	6.42	7.32	7.57
**$K_{Max}{}^B$**	4.4607	0.8154	1.92	6.24	2.38	2.94	4.55	5.67	5.98
**$K_{avg}{}^F$**	7.0109	0.7849	3.45	8.64	4.31	5.40	7.18	7.98	8.27
**$K_{avg}{}^B$**	5.7003	2.6020	2.65	85.05	3.19	4.03	5.62	6.92	11.17
**$Dz_{Max}{}^F$**	38.2438	26.2939	3.96	214.19	7.61	10.84	31.44	91.87	144.13
**$Dz_{Max}{}^B$**	84.8231	55.1712	-105.20	454.28	17.65	24.90	71.23	188.67	283.13
**NPtsR**	0.3388	0.2643	0.06	2.58	0.09	0.13	0.27	0.76	1.61
**Suspect keratoconus**									
**SI^F**	0.7707	0.4717	-0.54	2.57	-0.42	0.20	0.70	1.71	2.40
**SI^B**	0.2114	0.1455	-0.18	0.88	-0.11	0.02	0.19	0.51	0.72
**CSI^F**	0.6688	0.6608	-0.55	2.90	-0.48	-0.16	0.54	2.05	2.85
**CSI^B**	0.2235	0.1934	-0.21	1.00	-0.19	-0.05	0.19	0.63	0.98
**EI^F**	0.9442	1.0708	0	5.91	0	0	0.66	3.28	5.40
**EI^B**	1.4394	1.2846	0	6.77	0	0	1.16	3.94	6.68

**RMS^F**	3.9458	1.5018	1.30	9.23	1.51	2.10	3.60	7.22	9.02
**RMS^B**	7.2752	2.6379	2.36	16.15	2.75	3.70	7.05	12.26	16.05
**Thk_{MIN}**	506.2	37.6	406	589	410	437	508	570	587
**SI^{Thk}**	9.0688	5.4483	-9.84	31.30	-8.07	-0.12	8.93	17.69	25.57
**%TI**	0.1595	1.6933	-6.22	6.79	-4.56	-1.95	-0.20	3.21	5.88
**%EpiTI**	-0.5497	0.7257	-1.02	1.00	-1.02	-1.00	-0.95	0.99	1.00
**$K_{Max}{}^F$**	7.4171	0.2850	6.73	8.01	6.75	6.94	7.41	7.93	8.01
**$K_{Max}{}^B$**	5.9715	0.3222	4.63	6.83	4.78	5.42	6.00	6.45	6.76
**$K_{avg}{}^F$**	7.6783	0.3033	7.01	8.39	7.07	7.20	7.64	8.22	8.38
**$K_{avg}{}^B$**	6.3931	0.2996	5.52	7.12	5.59	5.95	6.36	6.90	7.11
**$Dz_{Max}{}^F$**	7.2284	2.9265	2.53	18.64	2.78	3.62	6.40	12.97	17.50
**$Dz_{Max}{}^B$**	14.7557	6.7643	4.09	38.45	4.66	6.13	13.35	27.18	37.71
**NPtsR**	0.8605	0.5105	0.11	2.49	0.12	0.27	0.73	1.85	2.46
**Myopic post-op**									
**SI^F**	0.2931	0.7239	-3.85	12.25	-1.23	-0.68	0.28	1.24	2.18
**SI^B**	0.0286	0.0944	-0.82	1.04	-0.17	-0.10	0.03	0.16	0.29
**CSI^F**	-0.8319	1.2698	-9.23	2.52	-5.00	-3.03	-0.69	0.94	1.76
**CSI^B**	0.1256	0.1209	-1.59	0.81	-0.10	-0.03	0.12	0.31	0.43
**EI^F**	0.3016	0.8493	0	18.57	0	0	0	1.53	3.29
**EI^B**	0.1435	0.4796	0	11.26	0	0	0	0.78	1.82
**RMS^F**	5.3935	2.5851	1.11	30.93	2.14	2.75	4.83	9.53	15.36
**RMS^B**	5.6793	6.2527	2.21	194.32	2.55	3.19	4.97	8.79	17.79
**Thk_{MIN}**	464.6	44.6	306	619	358	390	465	539	563
**SI^{Thk}**	4.4979	7.1260	-64.34	65.30	-13.13	-5.51	4.51	15.41	20.60
**%TI**	4.2098	2.5991	-9.39	16.44	-0.95	0.36	4.15	8.57	10.53
**%EpiTI**	-0.7687	0.5248	-1.19	1.00	-1.05	-1.01	-0.96	0.90	1.00
**$K_{Max}{}^F$**	7.4171	0.3792	5.39	8.57	6.31	6.76	7.44	8.00	8.29
**$K_{Max}{}^B$**	6.1033	0.4297	1.49	7.10	4.64	5.39	6.15	6.64	6.85
**$K_{avg}{}^F$**	8.3713	0.4513	7.37	11.43	7.59	7.77	8.31	9.14	9.84
**$K_{avg}{}^B$**	6.5729	1.1779	5.63	50.08	5.82	6.07	6.53	7.05	7.24
**$Dz_{Max}{}^F$**	9.5632	4.7148	2.83	54.92	3.76	4.76	8.62	16.87	27.14
**$Dz_{Max}{}^B$**	10.0760	11.1676	2.31	336.73	3.65	4.79	8.80	17.14	32.20
**NPtsR**	1.8879	0.4806	0.26	3.00	0.86	1.04	1.92	2.63	2.81
**Abnormal**									
**SI^F**	0.7193	4.3397	-18.00	22.95	-9.91	-5.59	0.37	9.04	18.07
**SI^B**	0.1829	0.8774	-4.62	4.04	-2.91	-0.92	0.09	1.63	3.03
**CSI^F**	0.2077	5.0762	-14.97	23.20	-10	-6.76	-0.09	10.98	19.74
**CSI^B**	0.1197	0.8678	-4.53	3.00	-2.82	-1.23	0.14	1.59	2.49
**EI^F**	3.6587	7.5852	0	71.67	0	0	0.21	18.85	35.80
**EI^B**	1.9247	3.9919	0	31.17	0	0	0	11.16	18.84
**RMS^F**	21.2811	17.6168	2.43	148.13	3.14	4.59	16.50	51.70	89.05
**RMS^B**	39.5106	64.7475	3.59	1257.02	4.19	5.66	30.53	89.83	228.75
**Thk_{MIN}**	496.0	102.5	72	923	175	317	509	639	756
**SI^{Thk}**	5.3713	25.3369	-221.41	129.51	-80.87	-24.27	5.50	41.55	65.74
**%TI**	5.3938	4.8708	-6.68	26.03	-2.02	-1.28	4.98	14.42	19.16
**%EpiTI**	-0.0724	0.8995	-1.19	1.00	-1.09	-1.00	-0.56	1.00	1.00
**$K_{Max}{}^F$**	5.8320	1.1122	2.62	7.99	3.24	3.96	5.85	7.46	7.85
**$K_{Max}{}^B$**	4.6691	0.9370	2.28	8.17	2.63	3.07	4.63	6.13	6.60
**$K_{avg}{}^F$**	7.7791	0.9666	4.41	15.27	5.37	6.59	7.65	9.46	11.53
**$K_{avg}{}^B$**	7.8795	8.7506	4.43	152.36	4.95	5.50	6.35	13.69	32.09

**Dz_{Max}^{F}**	40.0429	32.7342	1.87	251.11	4.61	7.26	30.34	101.50	158.39
**Dz_{Max}^{B}**	64.2505	55.0064	-99.78	538.30	6.77	9.98	53.17	161.34	278.61
**NPtsR**	1.6970	0.6331	0.15	3.50	0.27	0.61	1.71	2.71	3.15

Supplemental Table 5. Descriptive statistics in terms of mean (with standard deviation) and percentiles for each endpoint and each group.
